# Improved Patient Outcomes with Electrocauterization Following Wedge Resection and Curettage for Ingrown Toenails: A Prospective Comparative Study

**DOI:** 10.1055/s-0043-1777280

**Published:** 2024-02-29

**Authors:** Marzouq Amarin, Raed Al-Taher, Khaled Daradka, Amal Ibraheem Abd al Qader Abu Harb, Rawan Abd AlMohsen Mohammad Habashneh, Nadwa Basem Bustami, Yazan Hijazein, Hiba Hadadin, Sondos Wa'el Sa'dat Al-Najjar

**Affiliations:** 1 Department of General Surgery, Jordan University Hospital, The University of Jordan, Amman, Jordan; 2 Department of Pathology and Forensic Medicine, Jordan University Hospital, The University of Jordan, Amman, Jordan

**Keywords:** ingrown nails, electrocautery, curettage, excision, onychocryptosis

## Abstract

**Background** Ingrown toenail is a common condition that results in chronic pain, recurrent infections, and difficulty in performing daily activities. Our aim is to compare two surgical methods for the treatment of ingrown toenails: wedge resection with curetting versus wedge resection curetting followed by electrocauterization of the nail bed.

**Methods** A prospective, comparative study that included 130 patients with ingrown toenails. All patients had stage II or III disease. We divided the participants into two groups according to the type of surgery and all patients were followed up for 6 months. The outcomes measured were the incidence of postoperative bleeding and infection, recovery time, patient satisfaction, and recurrence rate 6 months after surgery.

**Results** Of the 130 patients included, 59 (45.4%) underwent excision and curetting of the nail matrix (group 1) and 71 (54.6%) underwent excision, curetting, and electrocauterization of the nail matrix (group 2). The postoperative infection rates were 20.3 and 4.2% in the first and second groups, respectively ( *p* = 0.004). Patient satisfaction was 76.3% among the first group, while 91.5% of patients in the second group were satisfied with the results of surgery. Six months postoperatively, recurrence rates were 25.4 and 4.2% in the first and second groups, respectively ( *p* = 0.001).

**Conclusion** Wedge excision and curettage, followed by electrocauterization of the ingrown toenail is a safe treatment modality with a high success rate, that is evident by a lower recurrence rate, and greater patient satisfaction, with no effect on postoperative pain score or recovery time.

## Introduction

An ingrown toenail significantly affects the quality of life through chronic pain and recurrent infections, as well as impairing daily life activities. Although multiple treatment modalities have been proposed, recurrence rates have been proven to be lower with surgical treatment than with conservative treatment. 1 When selecting the best approach for treating ingrown toenails, it is important to prioritize methods that are effective in minimizing postoperative complications to facilitate rapid return to normal daily activities. In addition, low recurrence rates, minimal risk of postoperative infection, and acceptable cosmetic outcomes are key considerations.

The Winograd method is a well-known surgical procedure that has been used since 1927 to treat ingrown toenails. It comprises partial nail plate removal and curettage of the nail bed and germinal matrix. Such minimally invasive techniques have been developed over time, including wedge excision of the nail fold and total ablation of the germinal matrix via chemical or electrical matricectomy, to prevent recurrence. 2

Several studies have been conducted on common surgical techniques, demonstrating that the Winograd method for ingrown toenails results in high satisfaction, low recurrence, and low complication rates. The addition of electrocoagulation of the germinal matrix to the Winograd method could result in even lower recurrence rates while maintaining high patient satisfaction without increasing the risk of complications. 3

Ozan et al compared partial matricectomy with either curettage or electrocautery and concluded that both surgical methods are safe treatment modalities with high success rates. They also concluded that partial matricectomy, achieved using curettage, is superior to electrocautery in terms of inflammation and pain duration. The results were inconclusive because there were advantages and disadvantages for each, but wedge resection was always associated with a low recurrence rate, with acceptable rates of postoperative infection, duration of the procedure, and patient satisfaction. 4

Therefore, we conducted a prospective comparative study to compare two different surgical methods for the treatment of ingrown toenails: wedge resection with curetting of the ingrown toenail bed, and wedge resection and curetting followed by electrocauterization of the nail bed.

## Methods

### Study Design and Participants

This prospective comparative study was conducted between April 2018 and May 2020. The sample size was calculated using the formula of Kish (1965), and the required sample was 68. We included 130 patients with stage II or III ingrown toenail in which two surgical approaches for treating ingrown toenails were compared. We excluded patients who had previous ingrown toenail and presented with recurrence at the time of the study, or those who had bilateral ingrown toenails and preferred to do both sides during the same operation were excluded. Patients with diabetes mellitus, chronic renal failure, and those with chronic lower limb ischemia were excluded as well since such conditions may affect the outcome.

The participants were divided into two groups, each of which underwent a different surgical approach. The first surgical approach included two methods (without cauterization), and the second included three methods (with cauterization). Choosing the surgical approach was performed by considering the first surgical approach for patients presenting during odd months (January, March, May, July, September, and November) and the second surgical approach for patients presenting during even months (February, April, June, August, October, and December).

All the surgical procedures were performed by the same surgeon. The first group ( *n* = 59) underwent the first approach, which included a lateral skin-fold incision and wedge excision of the ingrown toenail, followed by curettage of the nail germinal matrix (wedge excision and one method of mechanical matricectomy). The second group ( *n* = 71) underwent the other approach, in which a lateral skin-fold incision, a wedge excision of the ingrown toenail, along with curettage of the nail germinal matrix were done, followed by electrocauterization of the nail germinal matrix.

A data collection tool was developed by the authors based on an intensive review of the literature and approved by three experts in the field. The tool consists of 10 sections that assess patient characteristics at baseline (i.e., age and gender) as well as postoperative complications, including pain score, recovery time, bleeding, recurrence rate, postoperative surgical site infection (defined as erythema, swelling, increased pain intensity), discharge from the site of surgery (malodorous, thick, yellowish, or greenish) within 1 month of surgery, and aesthetic satisfaction.

Data collection was performed by interviewing the patients in the clinic at baseline and second and tenth day postoperation, and then by a weekly follow-up phone call for 6 months to assess recurrence.

The study was approved by the Institutional Review Board of the institute. Written informed consent was obtained from each patient prior to participation. All methods were performed in accordance with relevant guidelines and regulations.

### Surgical Procedures

In both procedures, the toe was cleaned using povidone-iodine and a sterile drape was applied. A ring block with 2% plain lidocaine was injected at the base of the toe, and a tourniquet was applied (to reduce bleeding during the procedure) for an average of 8 minutes in both procedures. A small incision (3–5 mm) was made over the proximal nail fold using blade number 15 to expose the extended part of the nail plate under the proximal nail fold. Blunt dissection was performed to separate the edge of the nail plate from the nail fold using a fine blunt straight clamp, followed by twisting to avulse and deliver the ingrowing part of the nail plate. The ingrown portion of the nail plate (about one-fifth) was cut using tissue scissors. Matricectomy of the germinal matrix was performed using blade number 15, with extraction of the diseased matrix, followed by curettage using a curette, removal of the tourniquet, and irrigation with normal saline (0.9% NaCl). A single hemostatic polyamide 3–0 stitch was applied from the (lateral/medial) nail fold to the proximal nail fold.

In the second group, a further step was performed involving retraction of the (lateral/medial) nail fold, followed by cauterization of the nail germinal matrix using a bipolar cautery device (30 A, for 10 s), followed by removal of the tourniquet, irrigation with normal saline (0.9% NaCl), and a single hemostatic polyamide 3–0 stitch was applied from the (lateral/medial) nail fold to the proximal nail fold.

**Fig. 1:**
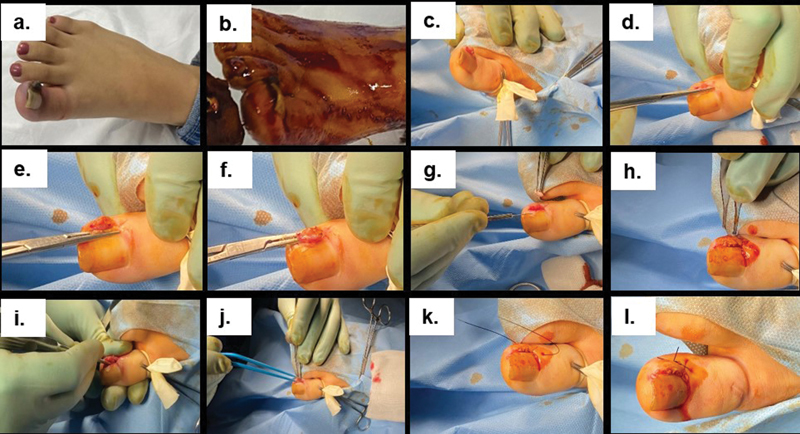
An illustration of the two different surgical methods for the treatment of ingrown toenails: (1) wedge resection with curetting of the ingrown toenail bed (without cauterization) ( **a–h** ). (2) Wedge resection and curetting followed by electrocauterization ( **a–j** ); and the last step of stitching ( **k, l** ).

**Fig. 2:**
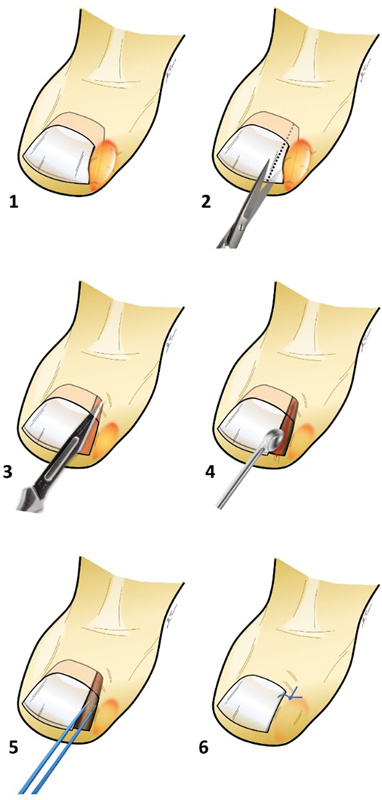
The figure shows a drawing of the two different surgical methods for the treatment of ingrown toenails: the first method, wedge resection with curetting of the ingrown toenail bed (without cauterization) ( **1–4** ). For the second method, the wedge resection, and curetting followed by electrocauterization ( **1–5** ); and the last step of stitching. 6

A sterile dressing with a fusidic acid ointment was applied. The wound was exposed after 48 hours in the clinic. The stitch was removed in the clinic on postoperative day 10. The steps involved in the two surgical methods are illustrated in Figs. 1 and 2 .

### Statistical Analysis

SPSS version 21 software was used for data analysis. Descriptive statistics including frequencies and percentages were computed. Frequency distribution was used for categorical variables, whereas means and standard deviations were used for continuous variables. The chi-square test was used to examine the correlation between two categorical variables, and the *t* -test was used to compare the means between two continuous variables. Statistical significance was set at *p* < 0.05.

## Results

Of the 130 patients included in the study, 59 (45.4%) were in the first group and 71 (54.6%) were in the second group. Table 1 summarizes the baseline characteristics of the study participants. The mean age of the patients was 26.3 and 27.2, in the first and second groups, respectively. The first group comprised 37.3% females and 62.7% males, while the second group comprised 46.5% females and 53.5% males ( Table 1 ).

**Table 1:** Patient characteristics at baseline ( *n* = 130)

	Without cauterization *n* = 59 (45.4%)	With cauterization *n* = 71 (54.6%)
**Age (years), mean ± SD**	26.3 ± 12.9	27.2 ± 13.9
**Gender**		
** Female**	22 (37.3)	33 (46.5)
** Male**	37 (62.7)	38 (53.5)

Abbreviation: SD, standard deviation.

Values represent the number of patients (percentage of the indicated group), unless stated otherwise.

Table 2 shows the outcomes measured at 2 and 10 days and at 6 months postoperatively. The mean postoperative pain score was 5.95 and 5.78 for group 1 and 2, respectively, while the mean recovery time was 10.37 days for the first group as opposed to 9.41 days for the second group with no statistically significant differences. An ingrown toenail recurred in 25.4% of the patients ( *n* = 15) in the first group and 4.2% ( *n* = 3) of the patients in the second group ( *p* = 0.000). Among the patients in the first group, 76.3% ( *n* = 45) were satisfied, 74.6% ( *n* = 44) would still undergo surgery if they had to do it all over again, and 79.9% ( *n* = 47) would recommend the surgery to someone else. In contrast, 91.5% ( *n* = 65) of the patients in the second group were satisfied with a significant *p* -value ( *p* = 0.016), 94.4% ( *n* = 67) would still undergo the surgery if they had to do it all over again ( *p* = 0.001), and 93% ( *n* = 66) would recommend the surgery to someone else ( *p* = 0.025). The incidence rates of postoperative infection were 20.3% ( *n* = 12) and 4.2% ( *n* = 3) in the first and second groups, respectively ( *p* = 0.004). No statistically significant differences were found between groups in terms of postoperative pain, recovery time, or postoperative bleeding.

**Table 2:** Outcomes on 2 and 10 days, and 6 months postoperatively

Variables	Without cauterization *n* = 59 (45.4%)	With cauterization *n* = 71 (54.6%)	*p* -value
Postoperative pain score (on a scale of 10), mean ± SD	5.95 ± 2.2	5.78 ± 2.3	–
Recovery time to return to work or school (days), mean ± SD	10.37 ± 4.9	9.41 ± 4.9	–
Postoperative bleeding (immediately and up to 48 hours after surgery)			0.104
Yes	7 (11.9)	3 (4.2)	
No	52 (88.1)	68 (95.8)	
**Postoperative infection (within 10 days of surgery, in the form of erythema, discharge, swelling, progressive increase in pain)**			0.004
Yes	12 (20.3)	3 (4.2)	
No	47 (79.7)	68 (95.8)	
Would you still have the surgery if you had to do it all over again?			0.001
Yes	44 (74.6)	67 (94.4)	
No	15 (25.4)	4 (5.6)	
Would you recommend the surgery to someone else?			0.025
Yes	47 (79.7)	66 (93)	
No	12 (20.3)	5 (7)	
Are you satisfied with the results of the surgery?			0.016
Yes	45 (76.3)	65 (91.5)	
No	14 (23.7)	6 (8.5)	
**Six** months follow-up for recurrence after surgery			0.000
Yes	15 (25.4)	3 (4.2)	
No	44 (74.6)	68 (95.8)	

Abbreviation: SD, standard deviation.

Values represent the number of patients (percentage of the indicated group), unless stated otherwise.

## Discussion

Ingrown toenails or (onychocryptosis) is a painful problem that affects people of all ages, particularly young people, and may become chronic if not treated. There are various causes of ingrown toenails and include incorrect clipping of nails, wearing tight shoes, obesity, as well as trauma to the toes and/or toenails, hyperhidrosis, fungal infection, and differential growth of toenails and toes during puberty. 5 The most frequent symptoms of ingrown toenails include pain, swelling, redness, and suppuration. Conservative treatment is preferred in mild cases. Surgery or conservative treatment can be used for stage 2 disease, characterized by deteriorating symptoms, drainage, and infection, whereas surgery is necessary for stage 3 disease, characterized by lateral wall hypertrophy. 6 Most previous research groups have studied three different surgical techniques. The first technique involves the classical wedge resection method with chemical matricectomy. 7 The second technique involved mechanical matricectomy (curettage vs. electrocauterization), 1 4 and the third technique involved the knot procedure. 5

Several studies have evaluated the Winograd method; the most widely used operative modality for ingrown toenails, and compared it to other techniques. One study evaluated the results of partial nail plate excision and curettage of the nail bed and matrix, and the results were in favor of Winograd technique with a recurrence rate of 7.9%. 2 Another study concluded that the Winograd method is superior in terms of patient satisfaction, recurrence, and complication rates. The authors concluded that the addition of electrocoagulation to the germinal matrix could result in a lower recurrence rate (0 vs. 6% in the Winograd method only) without increasing postoperative complications while maintaining a high satisfaction rate. 3 In contrast, a study comparing partial matricectomy with curettage and electrocautery concluded that both methods are safe and have high success rates. Partial matricectomy with curettage results in better outcomes in terms of inflammation and postoperative pain. 4 Another study compared wedge resection and wedge resection plus complete nail avulsion, and found no difference between the two methods, although wedge resection was cosmetically superior with a recurrence rate of 3.2 and 4.2%, respectively. 1 A recent study by Kim et al evaluated the outcomes of original Winograd procedure without wedge resection with electrocautery-aided matricectomy and showed a low recurrence rate (3.95%) and a high patient satisfaction. 8

However, when wedge resection was compared with NaOH matricectomy, both techniques were comparable in terms of recurrence rate, infection rate, and postoperative recovery, with a small advantage in the NaOH group. 9 Another long-term follow-up study compared surgical matricectomy with phenol chemical matricectomy. They found that surgical matricectomy resulted in lower recurrence rate (8.2 vs. 17.8%), whereas phenol chemical matricectomy resulted in better postoperative outcomes. 10 An effective treatment with a low recurrence rate was observed after germinal matrix destruction with cryotherapy, following partial toenail removal. 11 Studies comparing curettage and electrocauterization have reported that matrix removal using curettage with partial extraction of the nail is a simple and effective method for the treatment of ingrown toenails. 12 One study evaluated the outcomes of the Vandenbos procedure, and it was excellent regarding the recurrence rate, satisfaction rate, functional outcomes, recovery time, and complication rates. 13

Our study demonstrated that wedge excision followed by mechanical matricectomy in the form of curettage and electrocautery can result in decreased postoperative infection and recurrence rates, as well as increased patient satisfaction when compared with wedge excision and curettage alone. There were no statistically significant differences in recovery time, pain score, or postoperative bleeding. Romero-Pérez et al reported that treatment can be considered effective when there is no recurrence of the ingrown toenail for at least 6 months. 10

In our study, the recurrence rate was determined 6 months postoperatively, which could be a limitation; therefore, longer follow-up durations are needed to quantify recurrence rates better. Another limitation of the study was the patient selection process as they did not have an equal chance of treatment.

### Conclusion

A lateral skin-fold incision with wedge excision of the germinal matrix, followed by combining both methods of mechanical matricectomy in the form of curettage of the nail germinal matrix, followed by electrocauterization is a safe treatment modality with efficient destruction of the nail matrix and a high success rate. This is evident from the lower postoperative recurrence rates 6 months after surgery. In addition, this approach was associated with lower postoperative site infections, and greater patient satisfaction. Future studies should focus on comparing these techniques with chemical matricectomy.
